# The Kynurenine Pathway and Cancer: Why Keep It Simple When You Can Make It Complicated

**DOI:** 10.3390/cancers14112793

**Published:** 2022-06-04

**Authors:** Roumaïssa Gouasmi, Carole Ferraro-Peyret, Stéphane Nancey, Isabelle Coste, Toufic Renno, Cédric Chaveroux, Nicolas Aznar, Stéphane Ansieau

**Affiliations:** 1 Centre de Recherche en Cancérologie de Lyon, CNRS UMR5286, Inserm U1052, Université Lyon 1, Université de Lyon, 69373 Lyon, France; roumaissa.gouasmi@lyon.unicancer.fr (R.G.); carole.ferraro-peyret@adm.univ-lyon1.fr (C.F.-P.); isabelle.coste@lyon.unicancer.fr (I.C.); toufic.renno@lyon.unicancer.fr (T.R.); cedric.chaveroux@lyon.unicancer.fr (C.C.); nicolas.aznar@lyon.unicancer.fr (N.A.); 2 Department of Gastroenterology, Hospices Civils de Lyon, Lyon-Sud Hospital, Pierre Bénite, France and INSERM U1111—CIRI, 69365 Lyon, France; stephane.nancey@chu-lyon.fr

**Keywords:** NAD, kynurenine, tumor initiation and progression

## Abstract

**Simple Summary:**

The kynurenine pathway has two main physiological roles: (i) it protects specific organs such as the eyes and placenta from strong immune reactions and (ii) it additionally generate in the liver and kidney a metabolite essential to all cells of human body. Abnormal activation of this pathway is recurrently observed in numerous cancer types. Its two functions are hijacked to promote tumor growth and cancer cell dissemination through multiple mechanisms. Clinical assays including administration of inhibitors of this pathway have not yet been successful. The complex regulation of this pathway is likely the reason behind this failure. In this review, we try to give an overview of the current knowledge about this pathway, to point out the next challenges, and to propose alternative therapeutic routes.

**Abstract:**

The kynurenine pathway has been highlighted as a gatekeeper of immune-privileged sites through its ability to generate from tryptophan a set of immunosuppressive metabolic intermediates. It additionally constitutes an important source of cellular NAD^+^ for the organism. Hijacking of its immunosuppressive functions, as recurrently observed in multiple cancers, facilitates immune evasion and promotes tumor development. Based on these observations, researchers have focused on characterizing indoleamine 2,3-dioxygenase (IDO1), the main enzyme catalyzing the first and limiting step of the pathway, and on developing therapies targeting it. Unfortunately, clinical trials studying IDO1 inhibitors have thus far not met expectations, highlighting the need to unravel this complex signaling pathway further. Recent advances demonstrate that these metabolites additionally promote tumor growth, metastatic dissemination and chemoresistance by a combination of paracrine and autocrine effects. Production of NAD^+^ also contributes to cancer progression by providing cancer cells with enhanced plasticity, invasive properties and chemoresistance. A comprehensive survey of this complexity is challenging but necessary to achieve medical success.

## 1. NAD^+^-Fueling Pathways and Cancer Progression

The nicotinamide adenine dinucleotide (NAD^+^) and its reduced form (NADH) play a determinant role in maintaining cellular metabolism and cell survival. NAD^+^ serves as an electron acceptor essential for fatty acid oxidation, glycolysis and the TCA cycle. NADH is an electron donor oxidized during oxidative phosphorylation and is instrumental for ATP production. NAD^+^ additionally serves as a substrate in non-redox reactions for different families of enzymes, including the sirtuin deacetylases and the poly-ADP ribosyltransferase proteins (PARP) (for a recent review, see [1]). Sirtuin enzymes are essential for coordinating mitochondrial function, metabolism and aging, while PARP proteins ensure DNA repair and thereby preserve DNA integrity. Secreted NAD^+^ also interferes with immune and inflammatory responses (for a recent review, see [2]). NAD^+^ is generated from either vitamin B3 (with its three distinct forms: nicotinamide, nicotinic acid and nicotinamide riboside) or tryptophan (through its catabolism in the kynurenine pathway), both derived from the diet. It can also be reconstituted from nicotinamide, the product generated by NAD-consuming enzymes, through the salvage pathway. Under normal conditions, NAD^+^ is mainly dedicated to mitochondrial metabolic activity. During aging or after DNA damage, NAD^+^ is redirected to PARP and sirtuin proteins, which is essential to ensure genome integrity and orchestrate cell fate determination in such stress conditions [3].

**Figure 1:**
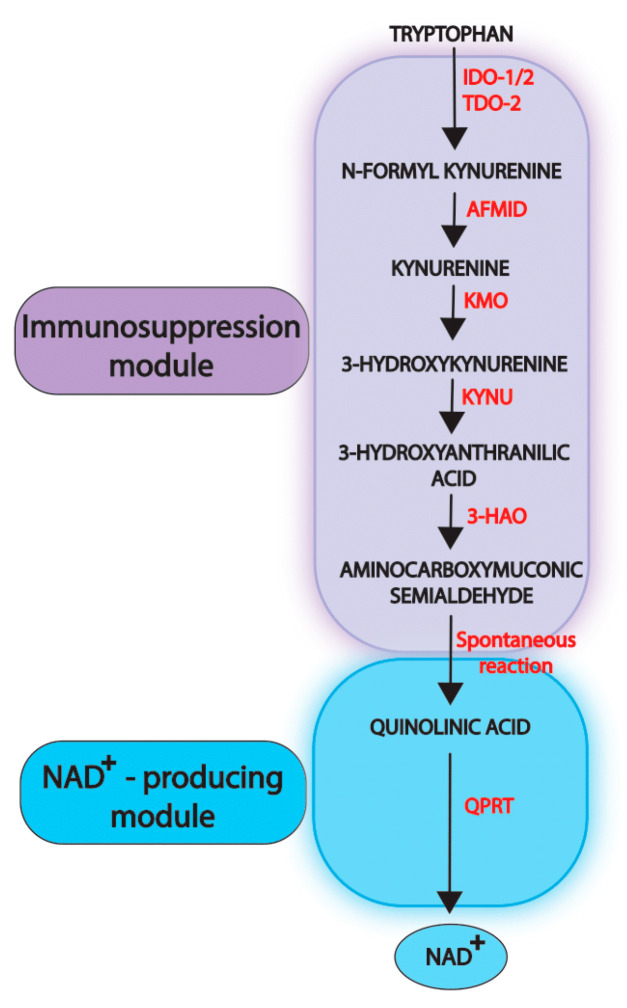
Schematic representation of the kynurenine pathway. Enzymes are written in red. IDO: indoleamine 2-3-dioxygenase, TDO2: Tryptophan 2,3-dioxygenase 2, AFMID: arylformamidase, KYNU: Kynureninase, 3HAO: 3-hydroxyanthranilate 3,4-dioxygenase, QPRT: quinolinate phosphoribosyltransferase. Immunosuppression and NAD^+^-producing modules are highlighted.

Cancer cells, in particular, are highly dependent on NAD^+^ to foster their metabolic reprogramming and to meet their higher demand for ATP. The salvage pathway constitutes the main source of NAD^+^ in cancer cells, and the nicotinamide phosphoribosyltransferase (NAMPT) is the bottleneck enzyme and the main regulator of intracellular NAD^+^ [4]. It is thus not surprising that *NAMPT* is overexpressed in cancer cells and that high *NAMPT* transcript levels are associated with a poor prognosis in several types of cancers ([5,6,7,8,9]). Different chemical drugs inactivating NAMPT have been generated and included in clinical trials in either advanced hematological or solid malignancies. Unfortunately, no significant anti-tumor benefits have been uncovered, and unexpected side effects resulting from combined toxicity and extracellular NAMPT activity have been reported [10]. Failure of NAMPT inactivation is also explained by the presence of alternative sources of NAD^+^ with the nicotinate phosphoribosyltransferase (NAPRT), which catalyzes the first step of NAD^+^ biosynthesis from nicotinic acid (the Preiss–Handler pathway), frequently active in cancer cells. Consistently, combined *NAMPT/NAPRT* high transcript levels have been reported as a significant poor prognostic factor in colon cancers [11]. As a second alternative source of NAD^+^, the activation of the kynurenine pathway, also constitutes a mechanism of escape from NAMPT-targeting therapies through the induction of quinolinic phosphoribosyltransferase-encoding gene *QPRT* [12,13]. The QPRT enzyme catalyzes the last and limiting enzymatic step of this metabolic pathway (Figure 1), converting quinolinic acid into nicotinic acid mononucleotide [14].

The contribution of the kynurenine pathway to cancer development has been largely documented with regard to the immunosuppressive properties of several of its intermediate metabolites. In the last decade, the oncogenic potential of these metabolites has been largely extended, and NAD^+^ production was additionally found to contribute to the protumoral properties of this pathway. We propose to review the current knowledge on these novel aspects, highlight the difficulties encountered to neutralize these deleterious effects and discuss future needs and potential alternative approaches.

## 2. Physiological Functions of the Kynurenine Pathway

The kynurenine pathway displays two essential functions (i) it fuels NAD^+^ production for the organism from tryptophan, and (ii) it dampens inflammatory responses in immune-privileged sites (including eye, brain, placenta and colon) through the production of immunosuppressive intermediate metabolites (kynurenine, anthranilic acid (AA) and 3-hydroxylanthranilic acid (3HAA)) [15]. NAD^+^ production is ensured in hepatocytes and renal proximal tubular cells, wherein the kynurenine pathway is active in its entirety, including QPRT ([16,17]). Oxidation of tryptophan, the first limiting step of this metabolic pathway, is catalyzed by the TDO2 (tryptophan-2,3-dioxygenase) or the IDO1 (indoleamine 2,3 dioxygenase 1) enzyme in hepatocytes or renal proximal tubular cells, respectively. A third enzyme, named IDO2 and related to IDO1, has recently been identified and is expressed in the liver, kidneys, brain, placenta and epididymis ([18,19]). The reduction of QPRT activity in the kidney was reported to be sufficient to significantly reduce NAD^+^ levels and sensitize patients to acute kidney injury (AKI). Restoration of NAD^+^ levels, achieved through oral administration of nicotinamide, overrides this effect, highlighting the preponderant role of de novo NAD production in kidney physiology [20]. The immunosuppressive function of the kynurenine pathway arises from a combination of effects. As numerous high-quality reviews have already addressed this point [21,22,23], this aspect will be briefly described. Consumption of tryptophan results in local depletion of this amino acid. Infiltrated T lymphocytes are sensitive to this modification, leading to the suppression of the mTOR pathway, the consequent activation of the GCN2-eIF2a signaling pathway and the subsequent cell-cycle arrest and anergy of infiltrating T lymphocytes. Kynurenine additionally behaves as a ligand and activator of the aryl hydrocarbon receptor (AhR) with pleiotropic consequences, including the suppression of innate immunogenicity of antigen-presenting cells and the activation of regulator T lymphocytes [21,22,23]. Lastly, the IDO1-kynurenine AhR axis upregulates PD1 expression in CD8 T cells providing an immune tolerant microenvironment for tumor cells [24].

In conclusion, the kynurenine pathway displays dual properties, including immunosuppressive functions and NAD^+^ production. The first function relies on the first steps of the pathway and the production of metabolic intermediates and can therefore be considered as a first “immunosuppressive module”. The second function relies on the activity of the QPRT, which thus orchestrates a second “NAD^+^-producing module” (Figure 1).

## 3. Dysregulation of the Kynurenine Pathway in Cancer

*TD02* is overexpressed in the primary colon, breast cancer, non-small cell lung cancer, ovarian and renal cell carcinomas as well as in brain metastases of malignant melanomas [25]. Its transcriptional induction is driven by inflammatory cytokines (such as interferon gamma IFNγ) and downstream NF-κB, C/EBPβ [26], as well as in response to AhR activation by kynurenine as a positive feedback loop [27]. *IDO1* is overexpressed and constitutes a poor prognostic marker in many tumors including laryngeal squamous cell [28], endometrial [29], cervical [30], oesophageal [31], gastric [32], colon [33], hepatocarcinoma [34] and melanomas [35]. Meta-analyses on a large number of clinical samples recently confirmed that a high *IDO1* transcript level could be considered a universal poor prognostic marker for solid tumors. Its high expression level is furthermore associated with tumor differentiation, distant metastasis and poor clinical stage [36,37]. Inflammatory cytokines such as interferon γ (IFNγ), interleukin 1β (IL1β) and tumor necrosis factor α (TNFα), through the JACK/STAT pathway, activate *IDO1* expression [38,39]. Furthermore, like *TDO2*, a high *IDO1* transcription level is sustained in cancer cells by an AhR-IL6-STAT3-driven positive feedback mechanism [40]. *IDO2* is dysregulated in a variety of cancers such as pancreatic, cervical and non-small cell lung carcinomas [41,42,43]. Its expression is induced by IFNγ, IL-10, prostaglandin E2 and LPS as well as by AhR [44].

The expression of other enzymes of the kynurenine pathway is also recurrently altered in cancers. Indeed *AFMID* was reported to be induced by c-MYC in colon cancers [45]. *KMO* is overexpressed in colorectal cancer patients, and high *KMO* expression predicts a higher risk of metastasis and decreases survival rate [46]. *KMO* is also amplified in 5 to 30% of breast cancers and promotes triple-negative breast cancer progression [47]. Contradictory results have been reported regarding the status of *KYNU* in breast cancers, depicting it either as a tumor suppressor gene or placing the enzyme downstream of the CD44-NF-κB signaling axis to promote breast cancer cell metastasis [48,49].

*QPRT* was identified as one the most upregulated genes in cancer [50]. The protein is aberrantly detected in breast, thyroid, colon, ovarian and cervical carcinomas, mela-nomas, gliomas and lymphomas (according to the Human Protein Atlas, [51]). *QPRT* ex-pression has so far mainly been associated with metastatic features and chemoresistance. In breast cancer patients, high *QPRT* expression is associated with a significant shorter recurrence-free and distant metastasis-free survival [52]. Gain- and loss-of-function ex-periments demonstrated that *QPRT* expression is associated with a gain of migratory and invasive properties. QPRT-mediated increment in cell migration and invasion relies on NAD^+^ production, purinergic receptor activation and downstream phosphorylation of the myosin light chain providing cells with an elongated morphology [52]. In high-grade gliomas, *QPRT* induction offers cancer cells the possibility to catabolize quinolinic acid produced by astrocytes into NAD^+^, thereby sustaining PARP and the associated base excision repair activities, and consequently rendering cancer cells chemoresistant [53]. NAD^+^-dependency of glioma cancer cells to overcome a combined histone deacetylase and proteasome inhibitor therapy was additionally highlighted in vitro, and in most cell lines it was associated with *QPRT* induction [54]. *QPRT* induction has also been associated with in vivo acquired resistance of small cell lung carcinoma cancer cells to EGFR inhibitors [55] and as one of the four genes included in a predictive signature of chemoresistance in colorectal cancers [56]. Modulation of *QPRT* expression in colon carcinomas is more complex and differs according to the microsatellite stability status of the tumors. In the microsatellite instable (MSI) tumor subgroup, *QPRT* was shown to be down-regulated and is currently considered to be part of the genetic MSI-specific signature [57]. Consistently, *QPRT* was shown to be methylated in the overlapping serrate tumor subgroup [58]. Conversely, *QPRT* is recurrently upregulated in microsatellite stable (MSS) colorectal cancers and its expression is associated with poor prognosis (TCGA database). 

## 4. Consequences of the Activation of the First Immunosuppressive Module

In cancer, genetic and epigenetic changes alter the activity of determinant enzymes or rewire oncogenic pathways, resulting in cell metabolic alterations. As a consequence of these alterations, accumulation of numerous metabolites provides cancer cells with selective advantages. The most extensively studied oncometabolites is the 2-hydroxyglutarate produced in response to *IDH1/IDH2* induction, known to impact the metabolic and epigenetic landscapes of cancer cells [59,60]. Several of the kynurenine pathway intermediate metabolites are now considered oncometabolites, particularly kynurenine. The recent determination of the metabolic landscape of cancer cell lines confirms that accumulation and secretion of kynurenine are correlated with *IDO* (mainly *IDO1*) and *TDO* transcriptional expression, with some cancer cell lines expressing both enzymes [61]. In vivo, the serum kynurenine/tryptophan ratio has been directly associated with either *IDO1* or *TDO2* expression and is correlated with disease progression in multiple cancer types, including breast, lung, colon and liver carcinomas [62,63,64,65]. Notably, while *IDO1* is overexpressed in gliomas, the kynurenine/tryptophan ratio is not predictive of disease progression in that tumor model [66], suggesting that the biological significance of this ratio is not universal. The oncogenic properties of kynurenine rely on pleiotropic effects depending in large part on its ability to activate AhR-responsive genes. Nuclear localization of AhR and induction of its downstream genetic program are recurrently detectable in a large panel of cancers. Furthermore, a TCGA analysis confirmed that a high score of the AhR-associated genetic signature is associated with poorer survival in bladder, cervical, glioblastoma, pancreatic, head and neck, and lung carcinoma, as well as in melanoma [67].

### 4.1. Paracrine Effects of AhR-Activation

Most studies focus on immunosuppressive properties of the kynurenine pathway, and in this context, as previously mentioned, the kynurenine-AhR axis is directly implicated in the suppression of innate immunogenicity of antigen-presenting cells and the activation of the regulator T lymphocytes [21,22,23]. Nonetheless, in the last decades, the kynurenine-AhR axis was shown to trigger tumor development in multiple ways. The kynurenine-AhR axis first promotes neoangiogenesis. The seminal observation that the knockout of *IDO1* in mice impairs oncogenic K-RAS-induced lung adenocarcinoma and the metastatic dissemination of xenografted breast cancer cells to the lung in association with a reduced density of vasculature in mice suggested a link between the kynurenine pathway and blood vessel formation [68]. Further analysis unveiled that *IDO1* induction by IFNγ results in the production of the proangiogenic IL6 protein. Activation of the kynurenine pathway thus behaves as a negative feedback mechanism to dampen IFNγ anti-angiogenic properties [69]. Mechanistically, both IDO1-driven tryptophan depletion and AhR activation contribute to cytokine production [70,71]. In liver cancer, the production of IL6 in response to the activation of TDO2-kynurenine-AHR signaling was also demonstrated to activate STAT3 and NF-κB/TIM4 signals to sustain cell proliferation, demonstrating additional protumoral properties of autocrine IL6 signaling pathways [65].

### 4.2. Intracrine Effects of AhR-Activation

Beyond its paracrine properties, kynurenine production was also shown to promote colon cancer cell proliferation through various mechanisms. In colorectal cells, c-MYC was demonstrated to activate tryptophan uptake and kynurenine production through the induction of a tryptophan transporter and *AFMID* expression [45]. The resulting kynurenine production, through AhR activation, was shown to display growth-promoting properties [45]. Consistently, specific depletion of IDO1 in intestinal epithelial cells was shown to reduce the progression of AOM-DSS intestinal carcinoma in mice [72]. In this study, the beneficial effect of IDO1 on cell proliferation was linked to β-catenin stabilization via a kynurenine-driven activation of the PI3K-AKT-GSK3β signaling [73].

Next, the constitutive activation of AhR was shown to promote the metastatic spread of breast, oral squamous, prostatic, gastric and colon carcinoma cancer cells [27,73,74,75,76,77]. High AhR protein level is correlated with lymph node metastases and/or poor prognosis in inflammatory breast and esophageal squamous cell carcinoma. A gain in migratory properties is generally associated with partial EMT, activation of the RhoA/ROCK1 and prostaglandin E2 production.

Lastly, rewiring tryptophan to kynurenine production also constitutes a novel chemoresistance mechanism. Indeed, in prostatic cancer cells, kynurenine accumulation in response to *TDO2* induction was shown to activate an AhR-cMYC-ABC transporter axis thereby providing cancer cells with chemoresistance. Disruption of this axis by c-MYC inactivation is sufficient to restore sensitivity [78]. Moreover, in triple negative breast cancers, AhR was more active in the ALDH1^high^ cancer stem cell subpopulation, and was reported to increase stem cell marker expression and properties, and chemoresistance [79]. Chemoresistance is also associated with intrinsic higher DNA and replicative stress signaling properties [80]. Bypass of DNA lesions in the replication fork, a process known as translesion DNA synthesis, is ensured by a subset of polymerases (among which hpol κ), enabling the bypass in an error-free or error-prone manner [81]. Overexpression of hpol κ in vitro or in animal models stimulates homologous recombination and promotes aneuploidy [82]. Interestingly, hpol κ expression has been correlated with advanced stages and identified as an independent poor prognostic factor for glioma patients [83]. Importantly, the TDO2-kynurenine-AhR axis was demonstrated to sustain hpol κ expression in glioma [84] and to thereby drive chromosomal instability. Indeed, TDO2 or AhR inactivation, similarly to the hpol k knock-down, reduces chromosomal instability in glioma cell lines, as assessed by micronuclei formation [84]. Combining TDO2 or AhR inhibitors with temozolomide has thus been proposed as an appealing alternative treatment for glioma patients [85].

**Figure 2:**
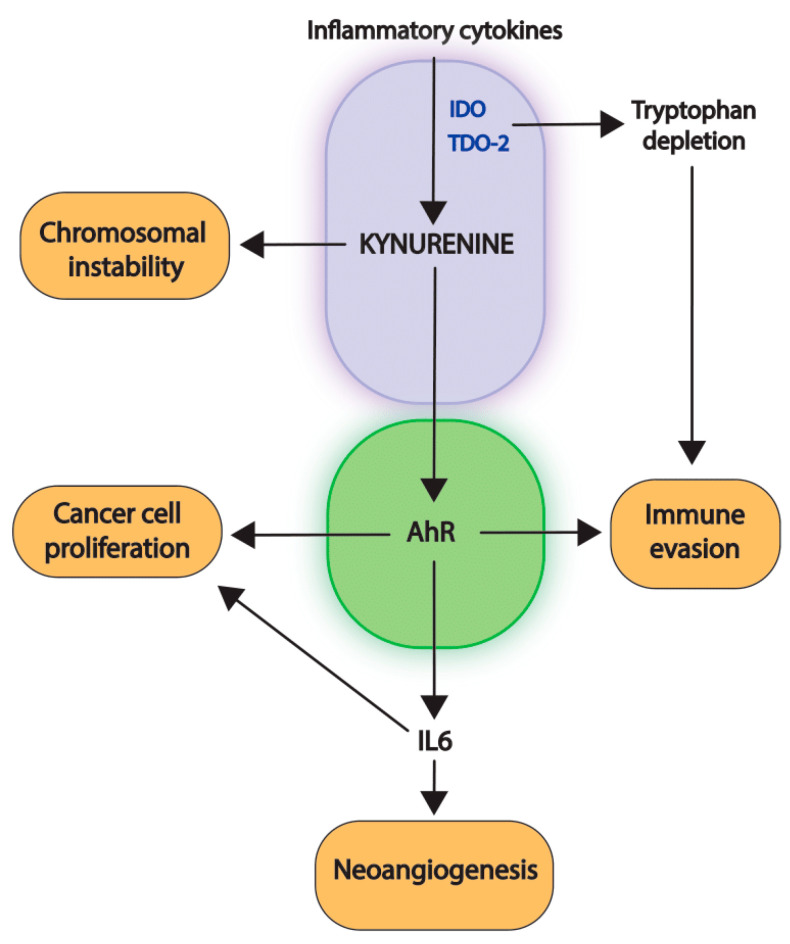
Pleiotropic kynurenine protumoral functions. Kynurenine production, resulting from *IDO* and/or *TDO* induction, leads to the AhR receptor activation to promote cancer cell proliferation, chromosomal instability, neoangiogenesis, immune evasion, metastatic dissemination and chemoresistance. AhR-independent functions of kynurenine also promote tumor growth. Tryptophan depletion, resulting from the kynurenine pathway activation, additionally contributes to the establishment of an immune tolerant environment.

In conclusion, the recurrent activation of the first module of the kynurenine pathway promotes tumor development by favoring tumor growth, genetic instability, immune evasion, metastatic dissemination and chemoresistance, mainly through the pleiotropic oncometabolite effects of kynurenine (Figure 2).

## 5. Contribution of the Second NAD^+^-Producing Module to Cancer Development

### 5.1. Induction of the Entire Kynurenine Pathway

The contribution of kynurenine pathway-driven NAD^+^ production in cancer progression still remains poorly explored. Bladder cancer progression was recently linked to the epigenetic silencing of the aldehyde oxidase gene (*AOX1*) by the methyltransferase EZH2 [86]. This loss-of-function rewires tryptophan catabolism to the kynurenine pathway, leading to the accumulation of kynurenine and NADP. As *AOX1* silencing enhances the bladder cancer cell transformation potential and increases their invasive properties through EMT invasion, activation of kynurenine might contribute to bladder cancer aggressiveness [86]. In line with this finding, the activation of the entire kynurenine pathway and subsequent production of NAD^+^ was recently shown to promote cancer cell dissemination in triple-negative breast cancers through purinergic receptor activation and downstream phosphorylation of the myosin light chain [52].

### 5.2. Consequences of the Inactivation of One or Both Modules

Conversely, inhibition of the kynurenine pathway in cells in which the pathway is entirely active (hepatocytes and renal tubular epithelial cells) also promotes tumorigenesis, the underlying mechanisms depending on the level at which the pathway is blocked and the consequences of it. In clear cell renal cell carcinomas (ccRCC), expression of *KMO*, *HAAO* and *QPRT* genes is recurrently and simultaneously downregulated. It was proposed that the expected accumulation of kynurenine favors tumor evasion [87]. Nonetheless, authors highlighted an accumulation of quinolinic acid (the substrate of QPRT) in tumors and demonstrated that QPRT depletion in ccRCC cell lines enhances their transformation potential and is associated with quinolinic acid accumulation [87]. Whether this metabolite directly drives this selective advantage and behaves as an oncometabolite remains to be addressed.

The investigation of the oncogenic potential of URI (the unconventional prefoldin RPB5 interactor) in liver cancer development also highlighted the tumor suppressive properties of the kynurenine pathway in this context. Its ectopic expression in hepatocytes simultaneously downregulated the expression of *TD02*, *AFMID*, *KYNU* and *HAAO* and consequently alleviated de novo production of NAD^+^, induced DNA damage and promoted tumor development. Subjecting mice to a nicotinamide riboside diet was sufficient to neutralize this oncogenic potential, strengthening the preponderant role of the kynurenine pathway in sustaining DNA repair activity. The correlation between URI expression and NAD^+^ depletion was also confirmed in human patients [88]. In summary, in this context, the inactivation of the entire kynurenine pathway led to a lack of its final product (NAD^+^) and this depletion promoted tumor development. In keeping with this conclusion, the *FMID* gene was recently shown to be modulated through RNA splicing during hepatoma cell carcinoma development in humans. The shift from complete and active variants to spliced and inactive variants occurred at the early stages of hepatoma carcinoma and was associated with *TP53* mutation. Ectopic expression of the full-length protein in hepatocarcinoma cell lines was sufficient to restore NAD^+^ production, reduce DNA damage and inhibit cell growth [89].

**Figure 3:**
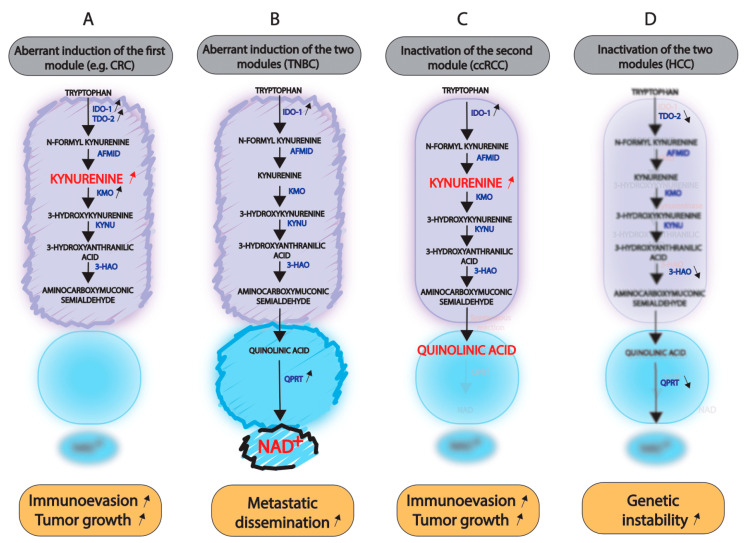
Different outcomes of kynurenine pathway alterations in cancer. Panel (**A**): the aberrant induction of the first module, as observed in colorectal carcinomas (CRC), leads to the accumulation of kynurenine, thereby promoting tumor growth and immune evasion. Panel (**B**): aberrant induction of the two modules, as observed in triple-negative breast cancers (TNBC), fuels cells with NAD^+^ and promotes cancer cell dissemination. Panel (**C**): Inactivation of the second module, as observed in clear cell renal cell carcinoma (ccRCC), leads to the accumulation of quinolinic acid and favors tumor development. (**D**): Inactivation of both modules, as observed in hepatomacarcinomas, prevents NAD^+^ production, favors genomic instability and, thereby, tumor development.

In conclusion, alteration of the kynurenine pathway can be direct or indirect, resulting from gain- or loss-of-function and epigenetic, genetic and splicing modifications. Its modulation is associated with tumor initiation and progression, metastatic spread of cancer cells, and chemoresistance. The consequences of kynurenine pathway alterations are also dictated by the relative activity of two oncometabolite- and NAD^+^-producing modules (Figure 3).

## 6. Targeting the Kynurenine Pathway: Difficulties and Challenges

The activation of the first module through the production of kynurenine oncometabolites and the resulting activation of AhR has several consequences, including promoting tumor growth, immune evasion, metastatic spread and chemoresistance. In light of these numerous deleterious effects, its potential therapeutic interest was obvious. IDO1 inhibitors, alone or in combination with chemotherapy, radiotherapy or immunotherapy, are currently under clinical trial [90]. However, the first Incytes’s phase III trial combining the IDO1 inhibitor epacadostat and a checkpoint inhibitor failed [91], highlighting the need to investigate all of the actors of the pathway further. Failure may have multiple etiologies, one of which being the redundant roles of IDO and TDO proteins. Combining inhibitors or targeting downstream enzymes constitute interesting alternatives. In this line, AhR also emerged as an appealing therapeutic target. Nonetheless, if AhR behaves as an oncoprotein in many tumors, it also displays tumor suppressive properties in other settings. As an example, *AHR* and *MYCN* expression were demonstrated to be inversely correlated in neuroblastoma, with an AhR staining correlated with tumor differentiation and favorable patient outcome. The group of patients with negative *AHR* and *MYCN* amplification showed the worst prognosis, whereas the group with *AHR* expression and normal *MYCN* expression showed the best survival [92]. Activation of receptors by kynurenine was shown to induce the differentiation of neuroblastoma cell lines, reduce their tumorigenic potential and, lastly, inhibit their metastatic potential through the induction of the tumor suppressor gene *KISS1* [92]. The choice to include AhR agonists or antagonists in therapies needs to be based on its exact functions in each tumor type and likely in each tumor subtype. Along this line, a first approach has consisted in defining in each tumor type an AhR-specific genetic signature to score it and assess its prognostic value. As an example, in lung cancer, a high AhR-associated genetic signature is associated with an unfavorable prognostic, implying the need to use antagonists [93]. Although attention has focused on the immunosuppressive properties of the pathway, its role in chromosomal stability, either by modulating PARP or polymerase activity, reshuffles the cards and underlines the interest in combining inhibitors of the pathway with DNA damage- and ROS-inducing conventional therapies. Interestingly, using the AhR-genetic signature to screen for efficient drug combinations has also been successful [93]. Defining the global status of the kynurenine pathway in tumors with a complete expression pattern of its enzymes and an intratumoral dosage of metabolites produced is also essential, not only to avoid treatment escape due to partial redundancies but also to focus on key enzymes. As an example, TDO2 inhibition impairs DNA damage tolerance in gliomas [94], while restoration of the immune response in multiple myeloma is achieved through KMO inactivation [95].

## 7. Targeting the Second Module of the Pathway as a Therapeutic Alternative

Analysis of the role of the kynurenine pathway in tumor development also highlights the contribution of the NAD^+^-producing module. NAD^+^ is essential for cancer cells to reduce the deleterious effects of ROS but also to provide them with plasticity and the capacity to adapt to hostile conditions. Fueling cells with NAD^+^ was, for example, defined as a determinant in melanoma to develop B-RAF inhibitor resistance and gain invasive properties [96,97]. The salvage pathway and its bottleneck enzyme NAMPT is the main NAD^+^-provider in cancer cells [4]. Aside from sustaining cancer cell proliferation, NAMPT additionally favors cancer cell dedifferentiation and, thereby, chemoresistance [98,99]. If the nicotinamide pathway is predominant in multiple cancer types, QPRT seems to take the lead in some of them. In gliomas and hepatocarcinomas, turning down the kynurenine pathway is sufficient to reduce deeply intracellular NAD^+^ concentration [53,87,88]. *QPRT* is also depicted as a stress-response gene [53], and its activity becomes predominant in stress conditions (e.g., inflammation) to meet the increased need for NAD^+^. As an example, in the THP1 monocyte cell line, immune tolerance is associated with a shift in NAD^+^ synthesis from the nicotinamide to the kynurenine pathway leading to a significant increase in nuclear NAD^+^ essential for SIRT1-driven epigenetic reprogramming [100]. Targeting QPRT in inflamed tumors could thus be efficient. It is reasonable to imagine that targeting both kynurenine and nicotinamide simultaneously, in light of their redundancies [12,13], in combination with conventional therapies, could be fruitful.

The activities of both modules are presumed to be interconnected by feedback mechanisms. A better understanding of the second module of this pathway and integrative analysis of the two modules over the course of tumor progression may help to decipher further their relative importance at different stages. A spatial and temporal expression of these enzymes is needed to obtain a complete overview. Future development of engineered mouse models in which *Ido*/*Tdo* and *Qprt* expression can be concomitantly or sequentially deleted in different cell contingents will also constitute essential tools to decipher the importance of the kynurenine pathway flexibility in maintaining tissue homeostasis in physiological and pathological conditions.

## 8. Non-Conventional Functions of the Kynurenine Pathway Enzymes

The last difficulty in delineating the exact contribution of the kynurenine pathway in pathologies is that several of the kynurenine pathway enzymes also display kynurenine-pathway independent functions. Generation of transgenic *IDO1* mice demonstrated that high *IDO1* expression levels, similar to those observed in inflamed mucosa, impact intestinal cell determination in favor of the secretory lineage. This modulation results in part from direct IDO1/AhR protein interaction and downstream modulation of the NOTCH signaling pathway [101]. KMO was also shown to enhance triple-negative breast cancer cell proliferation and migration. Although the enzyme was confirmed to be functionally active in these cells, its inactivation by chemical compounds did not impair its oncogenic potential [47]. KMO enhances the expression of the β-catenin downstream stemness genes *CD44*, *NANOG*, *OCT4* and *SOX2*. The underlying mechanism still remains elusive and could result from the ability of KMO to interact with β-catenin and GSK3β and, thereby, affect the phosphorylation and stability of β-catenin [47]. *QPRT* knock-down in SH-5YSY neuroblastoma cells impairs their ability to commit to a neuronal differentiation program and is linked with the induction of a genetic signature associated with autism [102]. The absence of modulation in kynurenine pathway metabolite concentration, and the failure to rescue the QPRT loss-of-function phenotype either by abrogating potential neurotoxic effects of the resulting accumulation of the QPRT-substrate quinolinic acid or with a NAD^+^ supply [102], let the authors conclude that this activity is also independent of the kynurenine pathway. Similar conclusions were drawn from QPRT-driven resistance to imatinib in large acute myeloid leukemia [103]. The pro-survival activity of QPRT may also be linked to its reported ability to interact and inactivate caspase-3 directly [104].

## 9. Conclusions

The kynurenine pathway is a complex metabolic signaling pathway integrating microenvironmental stress signals and hijacked by cancer cells to gain selective advantages. Production of oncometabolites and NAD^+^ promotes tumor growth, immune evasion and the spread of cancer cells and results from the activity of two flexible, interconnected and highly regulated modules. Deciphering their interplay and complementarity, resolving the complexity of their inter-regulation and redundancies, with an outcome obviously distinct in each tumor subtype and susceptible to fluctuate during tumor progression, is unquestionably a prerequisite to designing efficient therapeutic approaches in the near future.
